# Cell Type-Dependent Changes in CdSe/ZnS Quantum Dot Uptake and Toxic Endpoints

**DOI:** 10.1093/toxsci/kfv002

**Published:** 2015-01-19

**Authors:** Bella B. Manshian, Stefaan J. Soenen, Abdullah Al-Ali, Andy Brown, Nicole Hondow, John Wills, Gareth J. S. Jenkins, Shareen H. Doak

**Affiliations:** *Institute of Life Science, College of Medicine, Swansea University, Singleton Park, Swansea SA2 8PP, UK, ^†^Department of Medicine, Biomedical NMR Unit-MoSAIC, KU Leuven, B-3000 Leuven, Belgium and ^‡^Institute for Materials Research, SCaPE, University of Leeds, Leeds LS2 9JT, UK

**Keywords:** cell type, cellular uptake, nanoparticle, quantum dots, cytotoxicity, genotoxicity

## Abstract

Toxicity of nanoparticles (NPs) is often correlated with the physicochemical characteristics of the materials. However, some discrepancies are noted in *in-vitro* studies on quantum dots (QDs) with similar physicochemical properties. This is partly related to variations in cell type. In this study, we show that epithelial (BEAS-2B), fibroblast (HFF-1), and lymphoblastoid (TK6) cells show different biological responses following exposure to QDs. These cells represented the 3 main portals of NP exposure: bronchial, skin, and circulatory. The uptake and toxicity of negatively and positively charged CdSe:ZnS QDs of the same core size but with different surface chemistries (carboxyl or amine polymer coatings) were investigated in full and reduced serum containing media following 1 and 3 cell cycles. Following thorough physicochemical characterization, cellular uptake, cytotoxicity, and gross chromosomal damage were measured. Cellular damage mechanisms in the form of reactive oxygen species and the expression of inflammatory cytokines IL-8 and TNF-α were assessed. QDs uptake and toxicity significantly varied in the different cell lines. BEAS-2B cells demonstrated the highest level of QDs uptake yet displayed a strong resilience with minimal genotoxicity following exposure to these NPs. In contrast, HFF-1 and TK6 cells were more susceptible to toxicity and genotoxicity, respectively, as a result of exposure to QDs. Thus, this study demonstrates that in addition to nanomaterial physicochemical characterization, a clear understanding of cell type-dependent variation in uptake coupled to the inherently different capacities of the cell types to cope with exposure to these exogenous materials are all required to predict genotoxicity.

Photoluminescent, nanoparticulate, semiconductor quantum dots (QDs) have great potential for electronic, medical, and biological applications; they are proving to be particularly promising advanced imaging tools at the molecular and diagnostic level (Bagalkot *et al.*, 2007; Chen *et al.*, 2010; Fu *et al.*, 2009; Peng *et al.*, 2012; Wu *et al.*, 2003; Yang *et al.*, 2009). However, with increasingly widespread manufacture and use comes the risk of increased human and environmental exposure to, in some cases, significant numbers of these particles (Oberdorster *et al.*, 2005). When used in biomedical applications, these materials will likely be introduced into patients however disposal of consumer products containing QDs may also result in their release into the environment at high local concentrations, where they might accumulate and degrade (Kahru and Ivask, 2013; Scheringer, 2008). Our current knowledge of the potential health effects of exposure to QDs is mainly derived from acute cytotoxicity studies, and the data generated suggest that QDs may exert adverse effects in the skin (Zhang *et al.*, 2008), lungs (Geys *et al.*, 2008; Jacobsen *et al.*, 2009), gastrointestinal tract (Wang *et al.*, 2008), and other tissues (Soenen *et al.*, 2012; Tang *et al.*, 2008). The debate surrounding the potential toxicity of QDs still persists; for instance, no toxicity could be found in a pilot study on non-human primates (Ye *et al.*, 2012). Yet, it has been suggested that QDs are not excreted efficiently, thus, exposure could potentially lead to long-term health problems (Sealy, 2012). Furthermore, several studies have reported problems in correlating *in-vitro* to *in-vivo* findings thus more factors, such as nanoparticle (NP) dosing should be considered (Tsoi *et al.*, 2013; Yong *et al.*, 2013). It is also becoming increasingly apparent that any observed biological findings must be carefully correlated with the physicochemical properties of the QDs, as the many variations in chemical composition, structure, coating agents, and sizes make it very hard to derive general conclusions on toxicity (Singh *et al.*, 2009; Ye *et al.*, 2012).

Among recent findings the intrinsic ability of different cells to take up and process nanomaterials differently, thus potentially resulting in varying toxicity profiles, has been receiving increased attention. Although some studies have shown that CdSe/ZnS QDs can cause cytotoxic damage at specific exposure concentrations (Soenen *et al.*, 2012), whether this is true for all cell types remains an area of limited understanding. There are a small number of studies indicating that QDs do have some capacity for inducing DNA damage (Aye *et al.*, 2013; Choi *et al.*, 2012; Ju *et al.*, 2013), however, often the cell lines used in these studies were cancer derived that may be more resistant or sensitive to DNA damage and therefore may not be wholly representative of the *in-vivo* situation. Thus, to reduce the gap between *in-vitro* and *in-vivo* studies and to provide a better understanding of the toxicity results reported in *in-vitro* studies more research is needed to highlight the role of different cell types in governing the uptake and consequent potential genotoxicity following exposure to QDs. Furthermore, it has been shown that serum content in exposure media can affect NP uptake and hence mask the genotoxic potential of a class of NPs (Doak *et al.*, 2009) and this may be a confounding factor in many of the current QD reports. Another aspect that has been missed in previous studies is the role of time in the observed toxicity which has often has been limited to a maximum of 24 h. Thus, there is opportunity for investigations that systematically examine the genotoxic potential of QDs by associating uptake and DNA damage capacity with cell type while accounting for exposure times and varying serum conditions.

The aim of the present study was therefore to investigate variability in uptake and genotoxicity in 3 human cell lines with varying tissues of origin following exposure to QDs with different surface chemistries. The use of 2 QDs with similar chemical composition but coated with different functional groups (carboxyl vs amine) enabled additional consideration of the role of QDs surface functionalization in cellular uptake, cytotoxicity, and genotoxicity. Where cytotoxicity and/or genotoxicity was observed, underlying mechanisms were investigated, including the generation of reactive oxygen species (ROS), expression of inflammatory cytokines IL-8 and TNF-α, changes in mitochondrial membrane potential (MMP), and classification of DNA damage as aneugenic and/or clastogenic. This multiparametric approach allowed for an improved understanding of the role of the cell type in the observed genotoxic effects, particularly taking into consideration varying cellular growth characteristics as BEAS-2B and HFF-1 are adherent cells, while TK6 are suspension cells.

## MATERIALS AND METHODS

### 

#### Cell Culture

Human lymphoblastoid-B TK6 suspension cells, human bronchial epithelial BEAS-2B cells, and human foreskin fibroblast HFF-1 cell lines were applied in this study. All cell lines were purchased from ATCC (ATCC Cell lines Service) and were maintained in 75 cm^2^ flasks at a concentration of 1.5 × 10^5^ cells/ml. TK6 cells were cultured in Roswell Park Memorial Institute (RPMI-1640) medium supplemented with 1% 2 mM l-glutamine (Gibco, UK), and 10% horse serum (Gibco). BEAS-2B cells were propagated in Dulbecco’s Modified Eagle’s Medium (DMEM) in the presence of 10% foetal bovine serum (FBS; Gibco). HFF-1 cells required DMEM supplemented with 15% FBS. All cells were incubated in an atmosphere of 37°C and 5% CO_2_. For all the experiments, cells were seeded at 1.5 × 10^5^ cells/ml in culture medium containing reduced or full serum and allowed to settle overnight prior to treatment with 0, 2.5, 5, 7.5, 10, 15, and 20 nM dispersions of QDs for 1 or 3 cell cycles. These concentrations were selected according to the OECD guidelines which states that at least 4 concentrations which should cover a range of high toxicity to little or no toxicity should be used (Doak *et al.*, 2012). The highest concentration was selected based on previous results by Soenen *et al*. (2012). Experiments conducted at 2 cell cycles did not reveal significant differences to the 1 cell cycle results (data not shown).

#### Quantum Dot NPs

CdSe/ZnS core/shell fluorescent nanocrystals with amine- (Cytodiagnostics, Canada) and carboxyl- (Invitrogen, UK) functional ligands attached to the surface were used. The average diameter of each QD including its core and shell was 4–10 nm according to the manufacturer’s notes. The emission maxima of each QD were 585 and 665 nm for the carboxyl- and amine-QDs, respectively. Prior to cell exposure, carboxyl- and amine-QDs were suspended in water and vortexed for 30 s immediately prior to introduction into the cell cultures. The reduced serum concentration selected was based on optimization studies to identify the lowest serum content that could be applied for the experimental duration without altering cell growth parameters (data not presented). BEAS-2B and HFF-1 cells tolerated 2% serum while TK6 cells accepted 1% serum conditions. Cells were exposed to QDs for 1 or 3 cell cycles, where 1 cell cycle corresponded to 18 h for TK6 cells, and 24 h for both BEAS-2B and HFF-1 cells.

#### Physicochemical Characterization Studies

The hydrodynamic diameter, obtained by dynamic light scattering (DLS), and the zeta potential of the QDs were measured with a Malvern 4700 system (Malvern instruments Limited, UK) at 15 nM in water, RPMI-1640 medium with and without 1% or 10% horse serum, and DMEM with or without 2%, 10%, and 15% fetal bovine serum at 37°C. Data are presented as the average of 30 readings (10 readings per replicate).

The QDs were prepared for transmission electron microscopy (TEM) by placing a drop of suspended QDs onto a copper grid coated with a holey carbon support film (Agar Scientific Ltd) and plunge frozen in liquid ethane followed by freeze drying preserving the original features of the QDs (Hondow *et al.*, 2012). Images were subsequently captured. Images were collected by an FEI Tecnai TF20 FEG-TEM operating at 200 kV fitted with a Gatan Orius SC600A camera and an Oxford Instruments INCA 350 energy dispersive x-ray (EDX) system with an 80 mm^2^ X-Max SDD detector.

#### Cellular Uptake Studies

##### ImageStream analysis

Treated cells were harvested and FACS fixed (BD Biosciences, UK) for 30 min at room temperature. Samples were passed through the ImageStream imaging flow cytometer (Amnis Corporation) and fluorescence was measured at 488 and 633 nm. All experiments were conducted in duplicate and 5000 cells were acquired for each replicate. Data were analyzed using the Ideas v5 software (Amnis Corporation).

##### Transmission electron microscopy

For cellular uptake studies, samples were prepared as previously described (Hondow *et al.*, 2011). Briefly, the treated cells were harvested and placed in 2.5% glutaraldehyde fixative. Thin sections (>70 nm) were cut from the polymerized block using an ultra-microtome (Leica Microsystems, EM UC7). TEM was conducted as previously described (Hondow *et al.*, 2011) on a FEI Tecnai F20 operating at 200 kV and fitted with a Gatan Orius SC600A CCD camera for imaging and an Oxford Instruments X-Max SD detector for EDX analysis.

#### pH Effect on QD Stability

The effect of different pH levels on the fluorescence of these NPs was also investigated. QDs were incubated in 10% horse serum mixed with PBS and pH levels were adjusted to 7.4, 5.5, and 4.5. Particle suspensions were prepared at 2.5, 5, 5.5, 10, 15, and 20 nM concentrations in 100 µl total volume. Particles were incubated with the different media in black 96-well plates (Greiner Bio One BVBA, Belgium). All experiments were accompanied by a negative control and were conducted in triplicates. Fluorescence measurements were taken using the Omega multiwell plate reader (BMG Labtech, Belgium) on days 1, 2, 3, 4, and 5 post-preparation.

#### Cell Viability Assay

Cytotoxicity induced by exposure of the 2 QD types in BEAS-2B, HFF-1, and TK6 cell lines was determined according to their relative population doubling (RPD) as previously described (Singh *et al.*, 2012). All experiments were performed in duplicate with solvent-only negative controls and mitomycin-C (MMC) at 0.01 µg/ml was used as a positive control. Cell viability was considered significantly decreased when percent RPD was less than or equal to 50% (according to the OECD guidelines).

#### Cytokinesis-Blocked Micronucleus Assay

Gross chromosomal damage was quantified with the cytokinesis-blocked micronucleus (CBMN) assay, performed as previously described (Manshian *et al.*, 2013a) using a post-treatment cytochalasin-B protocol where cells were incubated for 24 h in full serum containing medium supplemented with 3 μg/ml cytochalasin B following treatment with QDs for 1 or 3 cell cycles. All experiments were performed in duplicate and MMC at 0.01 µg/ml was used as a positive control. Harvested cells were stained with 4′,6-diamidino-2-phenylindole (DAPI) and scanned on the Metafer automated scoring image analysis system (MetaSystems, Carl Zeiss Ltd). The frequency of micronuclei (MN) in 3000 binucleated cells per replicate was determined. As each concentration was performed in duplicate, the micronucleus frequency in 6000 binucleated cells in total per exposure concentration was assessed, which represents substantially enhanced sensitivity and statistical power over routine analysis which only requires scoring of 2000 cells per exposure concentration; OECD TG487.

#### Pancentromeric Staining

Slides prepared for the micronucleus assay were used for pancentromeric staining, however cells were fixed in 95% methanol for 10 min. Fluorescent *in situ* hybridization (FISH) was performed using a human pancentromeric probe labeled with FITC (Cambio, UK) and slides were analyzed under a Zeiss fluorescent microscope (Carl Zeiss, UK) at × 63 magnification. The presence of a centromeric signal was assessed in 100 MN (50 per replicate) present in binucleated cells. MN containing a fluorescence signal was classified as centromere positive containing a whole chromosome (aneugenic); while those lacking a fluorescently labeled region was centromere negative and therefore contained chromosome fragments (clastogenic).

#### ROS and MMP Analysis

ROS levels and MMP experiments were conducted in TK6 and HFF-1 cells as previously described (Soenen *et al.*, 2013). Briefly, 2 × 10^5^ cells/ml were seeded in black 96-well plates (Greiner Bio One, UK) and allowed to settle for 1 cell cycle after which they were treated with the QDs dispersed at 0, 2.5, 5, 7.5, 10, 15, and 20 nM concentrations for 4 or 24 h. Each experiment was conducted in triplicate and was accompanied with controls treated similarly but without addition of detection reagent or with QDs and reagent in the absence of cells verifying the induction of ROS in the cells due to the QDs and lack of QD interference with the ROS assay. All experiments were accompanied with positive control treatments of 0.33 M (1%) H_2_O_2_ for 2 h prior to incubation with 10 µM 5-(and-6)-chloromethyl-2′,7′-dichlorodihydrofluorescein diacetate, acetyl ester (CM-H_2_DCFDA; Molecular Probes, Invitrogen, UK) or 20μM JC-10 (Enzo Life Sciences, UK) for ROS and MMP experiments, respectively. Cells were washed twice with PBS and analyzed under an Omega microplate reader (BMG Labtech, UK) at 480 nm excitation with 540 nm emission (ROS analysis) or 520 and 590 nm emission (MMP assessment) according to the manufacturer’s recommendations. For MMP experiments, the data obtained were expressed as the proportion of damaged over healthy mitochondria (green/red).

#### Enzyme-Linked Immunosorbent Assay

TK6 and HFF-1 cells were seeded into T25 culture flasks at 1.5 × 10^5^ cells/ml in 10 ml total culture medium containing reduced and full serum. Following overnight incubation cells were treated with the QDs for 1 cell cycle, then the supernatant was collected and enzyme-linked immunosorbent assay (ELISA) (IL-8: Human CXCL8/IL-8 DuoSet and TNF-α: Human TNF-alpha DuoSet; R&D Systems, Abingdon, UK) was performed as per the supplier’s guidelines. All experiments were conducted in triplicate.

#### Statistical Analysis

All data are expressed as the mean ± standard deviation (SD). ImageStream results are represented as fluorescence intensity levels relative to untreated control cells and are expressed as the mean ± standard error of the mean. Micronucleus frequency was examined for significance with Fisher’s exact test, while ROS, MMP, and ELISA results were analyzed using one-way ANOVA.

## RESULTS

### 

#### Characterization of QD Physicochemical Properties

QD size distribution, morphology, crystallinity, zeta potential, and agglomeration status were investigated as a part of the physicochemical characterization study.

TEM analyses on the QDs in their primary-as purchased-state demonstrated that amine-QDs were generally 3–5 nm in diameter (Table 1), with evidence of crystallinity seen at higher magnifications while carboxyl-QDs were spherical and approximately 4–5 nm in diameter.

**Table 1.** Summary of the Physicochemical Characteristics of the QDs Investigated

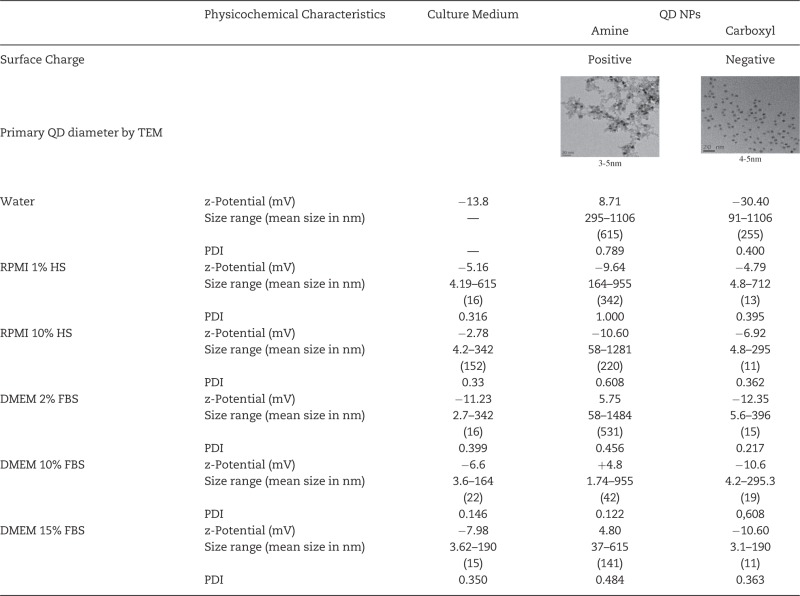

Surface charge, diameter according to TEM images of primary NPs, average zeta potential, and hydrodynamic diameter distribution of QD agglomerates in water, RPMI, or DMEM medium with reduced (1% or 2%) and full (10% or 15%) serum conditions is presented. DLS results are provided with their polydispersion index (PDI) values.

The hydrodynamic diameter for the QDs in the various solutions (Table 1) representing the size range of agglomerates in the serum containing media revealed that the amine-QDs formed larger agglomerates than the carboxyl-QDs (Table 1). Additionally, a clear variation in agglomerate size was noticeable between reduced and full serum conditions, where the QDs formed smaller agglomerates in full serum (FBS or HS) compared with reduced serum.

Overall, zeta potential measurements mainly revealed a low negative surface charge in the various dispersion media tested, indicating the potential for the absence of colloidal stability (Table 1). Amine-QDs demonstrated a slightly higher positive zeta-potential in water and in DMEM but this was near the neutral range. As anticipated, carboxyl-QDs showed a higher negative charge in water which was masked in the presence of media containing serum.

#### Cellular Uptake

Three, well established, genetically stable mammalian cells, TK6, BEAS-2B, and HFF-1, were used to examine QD uptake. Two image-based techniques were employed to investigate this parameter. ImageStream imaging flow cytometry and TEM. Quantitative measurements were attained from the analysis of ImageStream images. This approach allowed the quantitation of QD uptake which was coupled to direct identification and subcellular localization by TEM image analysis.

#### ImageStream Flow Cytometry

**FIG. 1.**
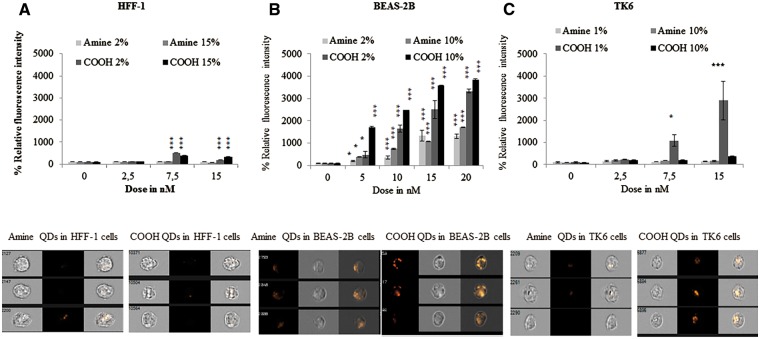
ImageStream cellular uptake analysis following QD exposure. A, HFF-1; B, BEAS-2B; and C, TK6 cells exposed to amine- and carboxyl-QD for 24 h in low serum or high serum containing media. Each graph is accompanied with representative results of cellular uptake images captured with ImageStream. Where appropriate, the degree of significance is indicated (*P < 0.05, **P < 0.01, ***P < 0.001).

Clear differences in relative intracellular fluorescence intensity were seen between the 3 cell lines exposed to each of the test QDs (Fig. 1). In general, fluorescence intensity, hence uptake levels, was much higher in BEAS-2B and TK6 cells compared with the HFF-1 cells. For BEAS-2B cells, clear concentration-dependent uptake could be seen for both amine and carboxyl-QDs, where this was not the case for HFF-1 cells. Carboxylated QDs demonstrated higher uptake than amine-QDs. For example, in BEAS-2B cells exposed to 15 nM of carboxyl-QDs, relative fluorescence intensity values of over 3000% were obtained, compared with 1100% for BEAS-2B cells exposed to an equivalent concentration of amine-QDs in full serum conditions. Carboxyl-QDs were readily taken up by all 3 cell lines with the highest uptake seen in BEAS-2B followed by TK6 and then HFF-1 cells. In TK6 cells this corresponded to a 2900-fold increase in cellular fluorescence in 1% serum conditions compared with the controls. Uptake of the same QDs was much less in 10% serum conditions (only 400-fold). No significant uptake was noted in these cells when exposed to the amine-QD. For both QDs serum conditions (reduced vs full) did not play a major role in cellular uptake levels except for TK6 cells exposed to carboxyl-QDs where significantly lower uptake was observed in full serum compared with reduced serum containing media (a > 20-fold drop of intensity between reduced and full serum) (Fig. 1C). Uptake levels in HFF-1 cells were substantially lower than the other 2 cell types and were not significantly different from negative controls except following exposure to carboxyl-QDs, which were significantly internalized at 7.5 and 15 nM concentrations in full and reduced serum conditions (Fig. 1A). Thus, the order of increasing cellular uptake based on cell line and QD surface coating type was BEAS-2B > TK6 > HFF-1 and carboxyl-QDs > amine-QDs, respectively.

#### Transmission Electron Microscopy

**FIG. 2.**
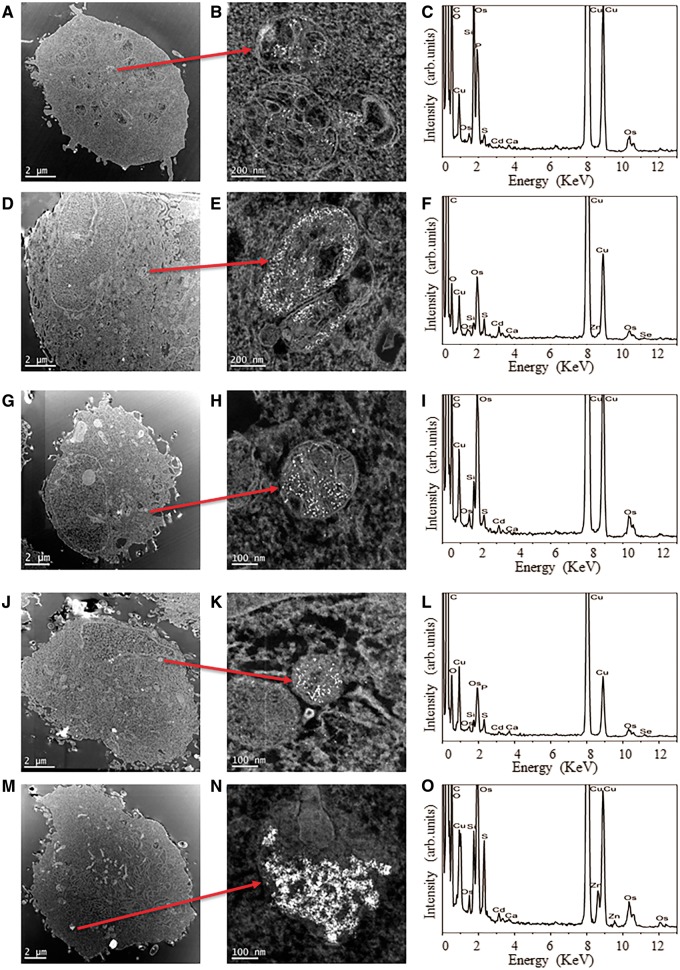
High angle annular dark field (HAADF) scanning transmission electron microscope (STEM) images confirming QDs uptake. (A) Carboxyl-QDs into TK6 cells accompanied with (B) higher magnification images of particles plus (C) assertion of particle composition by BF TEM EDX spectroscopy. No amine-QDs could be detected in TK6 cells using this technique (in line with ImageStream analysis). (D, E, F) Carboxyl- and (G, H, I) amine-QDs in BEAS-2B cells and (J, K, L) carboxyl and (M, N, O) amine-QDs uptake into HFF-1 cells.

Defining ultimate subcellular localization of NPs inside cells can only truly be achieved by TEM and thus, this technique was subsequently employed to ascertain the positioning of QDs inside the test cells. TEM images of BEAS-2B, TK6, and HFF-1 cells revealed the presence of carboxyl-QDs in all 3 cell types (Figs. 2A, 2D, and 2J). Amine-QDs were also identified within BEAS-2B (Fig. 2G) and HFF-1 cells (Fig. 2M). With respect to the carboxyl- and amine-QDs, in some instances (eg, Fig. 2M), large collections of QDs could be identified at low magnifications, however in all cases higher magnification imaging and elemental spectroscopy were undertaken to both confirm the presence of the QDs and also to determine the intracellular location (Figs. 2B, 2E, 2H, 2K, and 2N). The QDs could be found either free in the cytoplasmic space or localized in intracellular vesicles which appeared to be endosomes or lysosomes. TEM images suggested amine-QDs were present in larger agglomerates in vesicles within HFF-1 cells (Fig. 2N) compared with those in the BEAS-2B cells (Fig. 2H). Similarly, more carboxyl-QDs were detected in BEAS-2B cells (Fig. 2E) followed by TK6 (Fig. 2B) and HFF-1 cells (Fig. 2K), respectively, which correlates with the ImageStream data presented in Figure 1.

Cadmium was detected in all EDX analyses (Figs. 2C, 2F, 2L, and 2O), confirming the nanoparticulate features imaged were internalized QDs and not a sample preparation feature (eg, from the osmium tetroxide fixative) or an artefact (eg, signals due to the cellular environment, such as calcium). Elements from the TEM support grid itself (eg, copper and carbon) were also evident in the EDX spectra.

#### pH Effect on QD Degradation

**FIG. 3.**
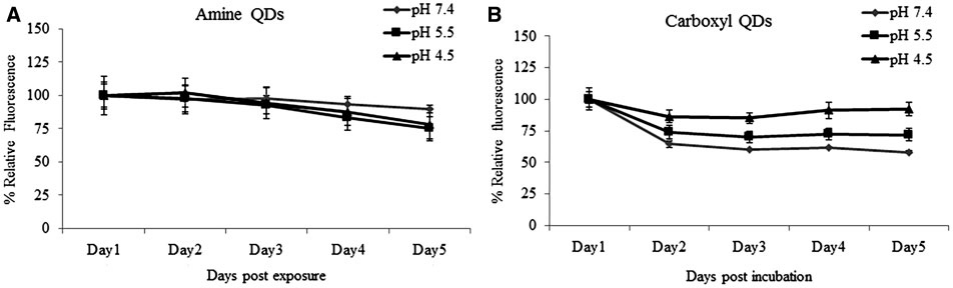
Effect of pH on QDot fluorescence intensity. Relative fluorescence intensity levels of 7.5 nM suspensions of amine- and carboxyl-QDs at various pH values (7.4, 5.5, and 4.5) as a function of time. Data are presented as mean ± SD.

The 2 QD particles were incubated with media adjusted to different pH levels (7.4, 5.5, 4.5) for 1, 2, 3, 4, and 5 days and fluorescence intensity was analyzed. These experiments were conducted at 0, 2.5, 5, 7.5, 10, 15, and 20nM concentrations, however, the graph shown here presents only data for the 7.5 nM dose for the purpose of conciseness. Results revealed a sharp decline in fluorescence intensity with carboxyl-QDs starting at day 1 in all 3 pH media (Fig. 3A). Some decline in fluorescence was detected in the amine-QDs (Fig. 3B), however, this was not significant at any time point.

#### Cytotoxic Effects of QDs

**FIG. 4.**
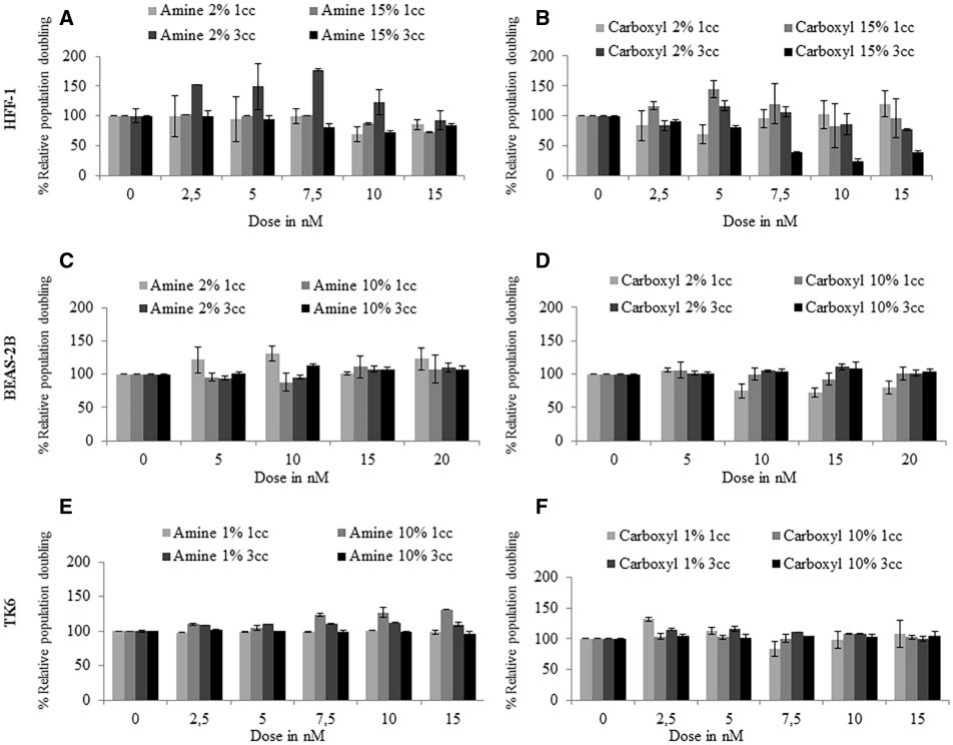
Cytotoxicity induced following exposure of human cells to QDs. (A, C, D) amine- and (B, D, E) carboxyl-QDs exposure to (A, B) HFF-1; (C, D) BEAS-2B; and (E, F) TK6 cells for 1 and 3 cell cycles exposure times in full and reduced serum containing media. Data are presented as mean ± SD.

Results of the RPD analysis revealed that no significant cytotoxicity was observed in BEAS-2B cells exposed to carboxyl- or amine-QDs in the presence of 2% or 10% serum after 1 or 3 cell cycles (Figs. 4C and 4D). Exposing HFF-1 cells to carboxyl-QDs in full (15%) serum containing media for 1 cell cycle induced notable cytotoxicity, which increased following 3 cell cycle exposures with significantly decreased cell viability (down to ≤ 38.5%) at concentrations ≥ 7.5 nM. This was however not the case in reduced serum experiments where no toxicity was observed (Figs. 4A and 4B). TK6 and HFF-1 cells suffered high levels of toxicity at concentrations higher than 15 nM (data not shown on graph) while BEAS-2B cells were able to tolerate concentrations of up to 20 nM (Figs. 4C and 4D).

#### QD Genotoxicity

**FIG. 5.**
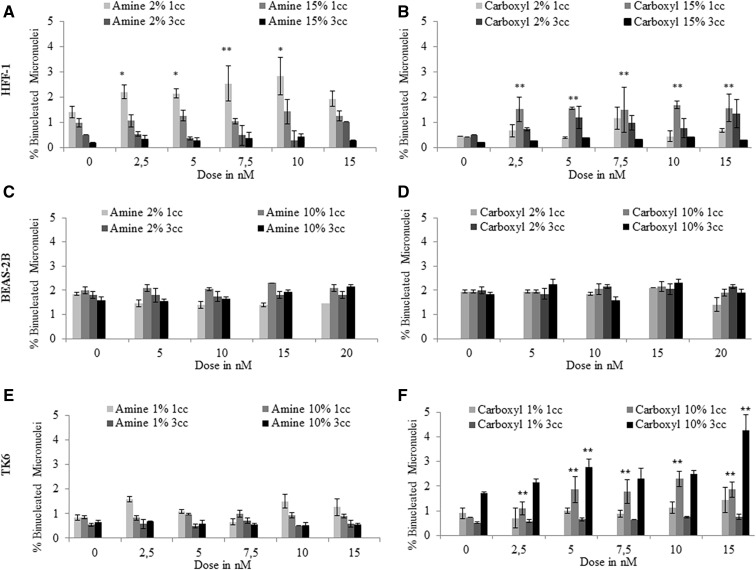
Micronucleus induction following exposure of human cells to QDs. (A) HFF-1, (B) BEAS-2B, and (C) TK6 cells exposed to amine- and carboxyl-QDs for 1 and 3 cell cycles time points in full and reduced serum containing media. Data are presented as mean ± SD. The MN frequency for the 0.01 µg/ml MMC positive control was 3.08 ± 0.44%, 2.3 ± 0.45%, and 5.2 ± 0.457% for TK6, HFF-1, and BEAS-2B cells, respectively. Where appropriate, the degree of significance is indicated (*P < 0.05, **P < 0.01, ***P < 0.001).

Chromosomal damage was analyzed by the CBMN assay, with MN scored in a minimum of 6000 binucleated cells per exposure concentration to enhance sensitivity of the test system. Of the 2 types of QDs examined, only the carboxyl-QD induced chromosomal damage in full serum containing media. The carboxyl-QDs resulted in a significant increase in MN frequency at several exposure concentrations in both TK6 and HFF-1 cells after 1 cell cycle exposures (Fig. 5). Prolonged exposure to carboxyl-QDs for 3 cell cycles in TK6 cells resulted in an increase in MN induction (Fig. 5F). HFF-1 cells showed a concentration-dependent increase in MN following exposure to amine-QDs up to 10 nM in media with reduced serum (Fig. 5A). No MN was detected in BEAS-2B cells exposed to either of the QDs (Figs. 5C and 5D). Therefore, no further analyses were conducted on these cell types due to the absence of any significant cytotoxic and genotoxic effects.

#### QD Genotoxicity Mechanisms

The mechanisms underlying the cytotoxic and genotoxic effects of the QDs were subsequently examined, focusing on the nature of the DNA damage, the effect of oxidative stress, and secondary mechanisms such as MMP (Δψ_m_).

**FIG. 6.**
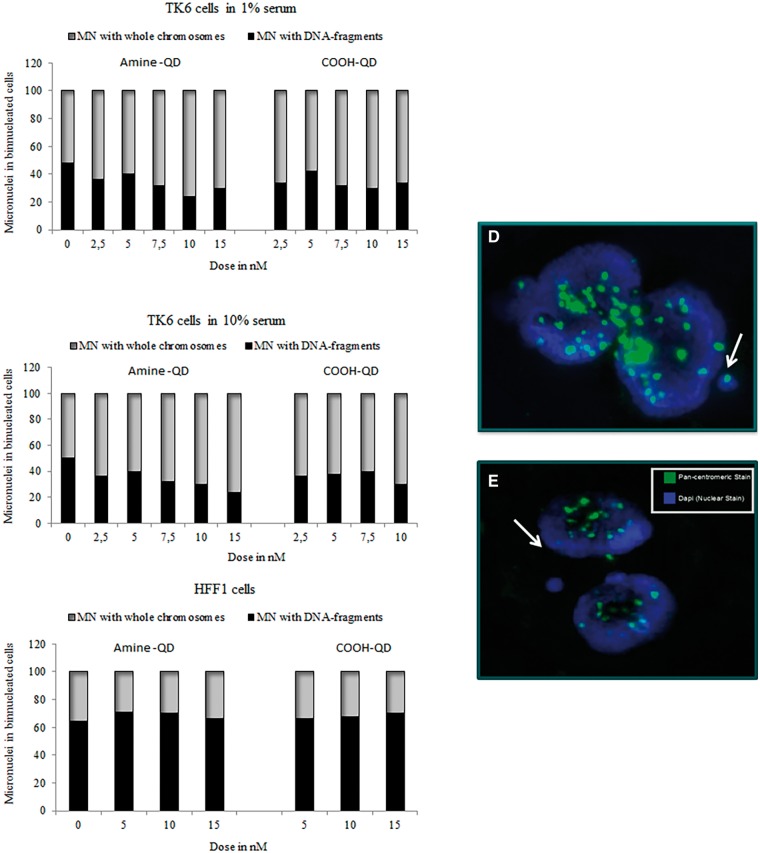
Ratio of micronuclei (MN) containing whole chromosomes (centromere positive) to DNA fragments (centromere negative) for TK6 cells exposed to QDs. Pancentromeric staining in TK6 and HFF-1 cells exposed to amine- and carboxyl-QDs for 24 h in (A) TK6 cells 1% serum; (B) TK6 cells 10% serum; and (C) HFF-1 cells 15% serum containing medium. (D, E) Representative fluorescence images of a binucleated TK6 cell with (D) or without (E) a centromere positive micronucleus (indicated by white arrows). Centromere-positive and centromere-negative MN were differentiated by the presence of bright yellow-green signal after pancentromeric antibody staining. Nuclei were counterstained with DAPI.

Pancentromeric staining was utilized to determine whether the gross chromosomal damage induced by the QDs was caused by clastogenic or aneugenic events. These experiments were conducted in both TK6 and HFF-1 cells in both full and reduced serum containing media. In TK6 cells amine-QDs showed a concentration-dependent trend of increasing aneuploidy (ranging from 50% to 76% MN containing whole chromosomes) induced in both 1% and 10% serum containing media (Fig. 6). This effect was less pronounced with the carboxyl-QDs. In HFF-1 cells pancentromeric detection was only performed in full serum conditions due to the absence of sufficient MN in the reduced serum conditions. Interestingly, in this cell line both QDs induced mainly clastogenic events (Fig. 6C).

**FIG. 7.**
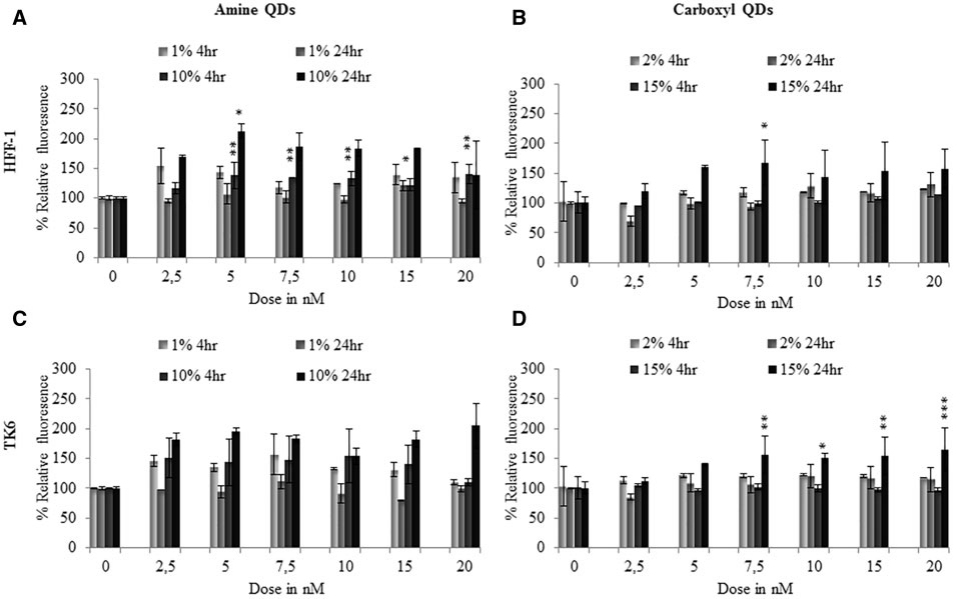
ROS induction in TK6 and HFF-1 cells treated with: (A, C) amine-, and (B, D) carboxyl-QDs for 4 or 24 h in full (dark gray) and reduced (light gray) serum conditions. Data are expressed as fluorescence intensity levels relative to untreated control cells and are represented as the mean ± standard error of the mean. The relative fluorescence intensity for the H_2_O_2_ positive control was 250 ± 50% and 200 ± 45% for the TK6 and HFF-1 exposed cells, respectively. Where appropriate, the degree of significance is indicated (*P < 0.05, **P < 0.01, ***P < 0.001).

**FIG. 8.**
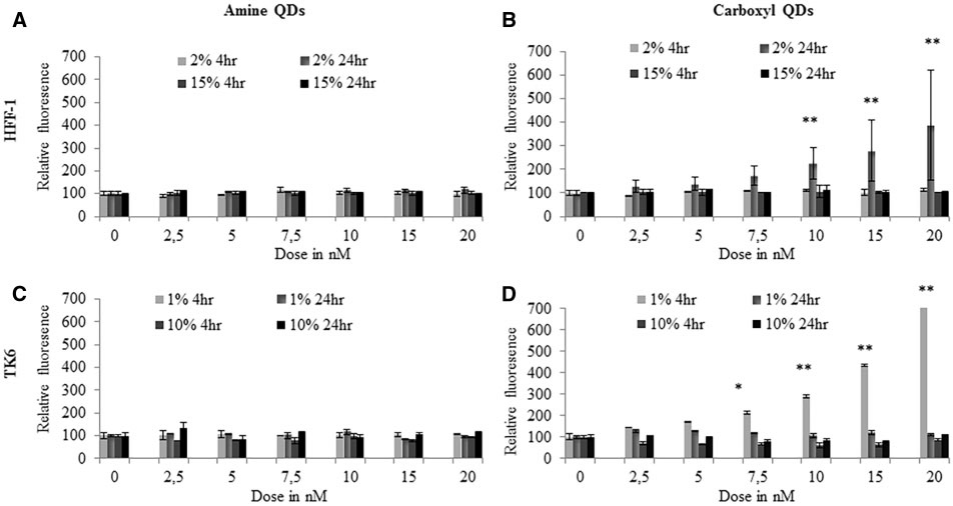
Mitochondrial damage induced following QD exposure. (A, B) HFF-1 and (C, D) TK6 cells following 4 and 24 h exposure to (A, C) amine-, and (B, D) carboxyl-QDs in low serum (light gray) and high serum (dark gray) conditions. Following uptake in healthy mitochondria, the green fluorescent JC-10 dye is converted into red clusters and the ratio of green over red mitochondria is used as a measure of the integrity of the mitochondria in the specific cell. Data are expressed relative to untreated control cells and are represented as the mean ± SD. The relative fluorescence intensity for the H_2_O_2_ positive control was 182 ± 22% and 130 ± 2% for the TK6 and HFF-1 exposed cells, respectively. Where appropriate, the degree of significance is indicated (*P < 0.05, **P < 0.01, ***P < 0.001).

The production of ROS was investigated due to its association with toxicity following exposure to certain NPs. However, only minimal cytoplasmic ROS was detected, mainly in HFF-1 cells exposed to the carboxyl-QD and TK6 cells exposed to amine-QDs (Fig. 7). With respect to mitochondrial membrane permeability, no observable effects were seen in HFF-1 or TK6 cells exposed to any QDs in full serum conditions (Fig. 8). In contrast, a significant and concentration-dependent increase in MMP was recorded in TK6 and HFF-1 cells treated with carboxyl-QDs in reduced serum containing media. This increase in MMP occurred after 4 and 24 h treatments in TK6 and HFF-1 cells, respectively (Fig. 8B and 8D).

The potential inflammatory effects of the QDs were also evaluated by determining the release of either IL-8 or TNF-α by TK6, BEAS-2B, or HFF-1 cells when exposed to amine- or carboxyl-QDs by means of specific ELISAs. The cells were exposed to the QDs for 24 h over a broad concentration range (0, 2.5, 5, 7.5, 10, or 15nM), but no increase in the level of excreted IL-8 or TNF-α could be observed for any QD type at any concentration compared with the level produced by untreated control cells (data not shown). These results were limited to the 2 cytokines investigated in this work which is not conclusive of the inflammatory state of these cell lines following exposure to the QDs in this study.

## DISCUSSION

The HFF-1, BEAS-2B, and TK6 cell lines are considered important targets for NP toxicity studies (Lai *et al.*, 2008; Li *et al.*, 2012; Nymark *et al.*, 2012) because they represent 3 major ports of exposure to NPs. However, the present investigation demonstrates that when exposed to the same NPs, each of these cells demonstrate clear differences in subsequent genotoxicity profiles. This could partly be related to the different tissue source of these cell lines being epithelial, fibroblast, and lymphoblastoid. The consequences of QD exposure were tested in full serum and reduced serum conditions, following acute and extended exposure durations to examine the role of cellular repair in overriding any observed damage. Nonetheless, QD toxicity was found to be highly dependent on the cell type under investigation.

Carboxyl- and amine-QDs with a similar core size, demonstrating a variable degree of agglomeration according to their surface chemistry, were used. Amine-QDs were found to agglomerate most extensively, whereas the carboxyl-QD agglomerates were relatively smaller. The degree of agglomeration also depended largely on the nature of the cell culture media and the amount of serum present. These differences in agglomeration appear to have led to significant variation in the resultant cellular interactions. The carboxyl-QDs formed the smallest agglomerates, and produced the most pronounced uptake levels in all 3 cell types. With respect to cellular uptake, imaging flow cytometry revealed that BEAS-2B cells exhibited the highest levels of QD internalization, followed by TK6 cells and then HFF-1 cells. Considering that quantifying fluorescence levels in the intracellular environment has its challenges as intracellular pH level alterations can affect their fluorescence quantum yield, thus, we examined the role of different pH levels on the QDs used. Our results showed that though changes in pH levels resulted in degradation in carboxyl and not amine-QDs but this did not affect its fluorescence intensity. However, it was clear from these analyses that carboxyl-QDs had a much higher innate fluorescence compared with amine NPs. For example, the fluorescence intensity of 7.5 nM carboxyl-QDs in pH 7.4 on day 1 was 259 810 compared with 51 061 in its amine counterpart at the same conditions. Even though there is a 5-fold difference in these values however this effect is not what we see in the ImageStream uptake results. Thus, the difference in cellular uptake stems from a combination of interrelated factors being the higher initial brightness of the carboxyl-QDs yet the reduced fluorescence intensity of these QDs in intracellular conditions, and differences in the cellular capacity for QD internalization. Moreover, even though the different cells had a different propagation time (18 h for TK6 and 24 h for the BEAS-2B and HFF-1 cells) but this difference was not big enough to be accounted for the difference noted in the cellular uptake. For example, in a study investigating proliferation of TK6 cells only 20% of the cells were found in G2/M phase following 6 h (Ren *et al.*, 2011) and in another study TK6 cells were evidenced to start the first doubling after 16 h (Noonan *et al.*, 2012). Although cellular uptake was observed with all 3 cell lines after 1 cell cycle this did not always result in significant cytotoxicity or genotoxicity.

This study demonstrated that uptake levels do not always correlate with the presence of toxicity since BEAS-2B cells demonstrated the highest level of uptake for both amine- and carboxyl-QDs yet they were most resistant to cytotoxic or genotoxic effects. The TK6 cells appeared to be the most sensitive, especially given the lower level of cell-associated QDs, indicating the cells were unable to tolerate even low levels of internalized QDs. The high internalization of carboxyl-QDs also resulted in the greatest induction of genotoxicity, oxidative stress, and mitochondrial damage all of which were found to show concentration-dependent relationships. All particles demonstrated clear toxicological differences depending on the presence of low or high serum conditions, which may be attributed to serum protein corona on the QDs affecting interactions between the QDs and the cellular membrane leading to alterations in the rate of internalization and in their intracellular effects.

Despite BEAS-2B proving to be the most resistant cell following exposure to QDs, in other studies, the same cells have shown some susceptibility to cytotoxicity. This is true for polystyrene NP where there was more obvious damage induced in BEAS-2B compared with macrophage, epithelial, and cancer cell lines (Xia *et al.*, 2008). Similarly, significant levels of chromosomal damage have been previously reported when BEAS-2B cells were exposed to single-walled carbon nanotubes (Manshian *et al.*, 2013b).

Differences in cellular response to QD exposure could partly be due to the mechanisms by which they impart cellular stress. Thus, to further understand the implications of QD exposure the generation of ROS was explored given its important role in toxicity generated from NPs and specifically QD exposure (Lewinski *et al.*, 2008; Soenen *et al.*, 2012). However, no significant induction of ROS was detected in any of the treatments here. These observations are different to ones previously reported by Soenen *et al*. (2012) where ROS induction was found for up to 20 nM exposure concentration to the same commercially available carboxyl-QDs as applied in the present investigation. This difference could be due to the very different cell types used (HUVEC, PC12, and C17.2 cells), with substantial differences in anti-oxidative capacity (Soenen *et al.*, 2012). It is also plausible that the ROS generated in these cells were not detectable with the assays used in this study. It is well known that quantitative analysis of ROS can often be hindered by the intracellular presence of high levels of thiyl or sulfinyl radicals formed by glutathione which along with other agents can lead to the scavenging of ROS (Cossarizza *et al.*, 2009). Although genotoxic effects were seen in the TK6 cells following exposure to QDs, the limited induction of ROS in TK6 cells correlates with the fact that largely aneugenic responses were detected (as oxidative stress typically induces clastogenicity) (Emerit *et al.*, 2000). Thus, the intrinsic homeostatic differences in varying cell types could be of particular importance in understanding the potential toxicity imparted by specific NMs.

Other factors that were determined to play a role in QD-induced toxicity in this study included QD surface charge, exposure media composition and serum content, plus the exposure duration. For example, here, HFF-1 cells demonstrated no cytotoxicity following exposure to amine QDs; yet significant cell death was observed at high concentrations when the cells were exposed to carboxyl-QDs for 3 cell cycles. When genotoxicity was considered, the carboxyl-QDs did not impart any chromosomal damage in the HFF-1 cells, while amine-QDs induced a significant induction of MN after 1 cell cycle which was absent following 3 cell cycles. This NP-dependent toxicity difference therefore highlights the importance of considering the physicochemical characteristics as well as other factors in such studies. Not only was this apparent when genotoxicity was considered, but was also responsible for different mechanistic processes underlying the cellular damage. For instance, carboxyl-QDs induced a significant concentration-dependent increase in MMP, while amine-QDs did not appear to cause any such change. These effects were only detected in reduced serum condition in both HFF-1 and TK6 cells. Consequently, this could suggest a role for the protein corona which might influence the uptake mechanics of these NPs (Monopoli *et al.*, 2012). It is well known that NPs bind to serum proteins at different extents depending on their surface charge. In 2 recent studies, negatively charged gold and iron oxide NPs bound more strongly to plasma proteins eliciting different biological responses to their positively charged counterparts (Deng *et al.*, 2013; Sakulkhu *et al.*, 2014). These findings might therefore explain our results where in general carboxyl-QDs were more readily taken up in reduced serum conditions compared with full serum media resulting in more pronounced cytotoxic and genotoxic consequences in these conditions.

In conclusion, QD-induced cytotoxicity and genotoxicity are strongly affected by a multitude of parameters including: (1) differences in cell type potentially resulting in varying surface area contact with the exposed material, in addition to inherent cellular differences in internalizing NPs and ability to cope with an exogenous insult; (2) the nature of the QD surface chemistry; (3) the degree of QD agglomeration in the presence of varying amounts of serum proteins; (4) differences in cell culture media composition; and (5) time of exposure. We suggest that these factors influence the degree of agglomeration and sedimentation of the particles that subsequently influence the level and nature of cell association. The latter translates itself in differences in cytotoxicity and genotoxicity that do not always directly correlate with the quantity of internalized material, but are also strongly influenced by the intrinsic cellular capacity for handling internalized foreign material, which is cell type dependent.

Thus, it is pertinent that future studies follow multiparametric approaches for studying NP-induced toxicity. Of particular importance is the consideration of multiple target organ-specific cell types in parallel to obtain a more complete understanding of the biological consequences of a specific nanomaterial exposure.
